# Digital setup accuracy for moderate crowding correction with fixed orthodontic appliances: a prospective study

**DOI:** 10.1186/s40510-024-00513-7

**Published:** 2024-04-08

**Authors:** Abdalrahman Mohieddin Kusaibati, Kinda Sultan, Mohammad Younis Hajeer, Nikolaos Gkantidis

**Affiliations:** 1 Department of Orthodontics, Faculty of Dentistry, University of Damascus, Damascus, Syria; 2 Department of Orthodontics and Dentofacial Orthopedics, University of Bern, Bern, Switzerland

**Keywords:** Computer simulation, Fixed orthodontic appliances, Dental model, Tooth movement technique, Accuracy, Three-dimensional imaging

## Abstract

**Objectives:**

To evaluate the accuracy of a semi-automatic 3D digital setup process in predicting the orthodontic treatment outcome achieved by labial fixed appliances.

**Subjects and Methods:**

Twenty-five adult patients (18 to 24 years old) with class I malocclusion and moderate crowding were prospectively enrolled and received treatment on both jaws through the straight-wire technique. Prior to treatment commencement, a semi-automatic digital setup simulating the predicted treatment outcome was performed for each patient through Orthoanalyzer software (3Shape®, Copenhagen, Denmark) to obtain the prediction model. This was compared to the final outcome model through 3D superimposition methods. Metric variables and inspection of color-coded distance maps were used to detect how accurately the digital setup predicts the actual treatment outcome.

**Results:**

The mean absolute distances (MAD) between the superimposed dental arches of the predicted and the final models were: 0.77 ± 0.13 mm following superimposition on the palate, 0.52 ± 0.06 mm following superimposition on the maxillary dental arch, and 0.55 ± 0.15 mm following superimposition on the mandibular dental arch. The MAD at the palatal reference area was 0.09 ± 0.04 mm. Visualization of color-coded distance maps indicated that the digital setup accurately predicted the final teeth position in a few cases. Almost half of the cases had posteriorly wider upper and lower dental arches and palatally/lingually positioned or inclined anterior teeth, whereas the rest still showed errors within 2–3 mm, distributed over the entire dental arches with no distinct pattern.

**Conclusions:**

The accuracy of semi-automatic prediction of the labial fixed appliance treatment outcome in Class I cases with moderate crowding is not yet sufficient. While average measures showed deviations less than 1 mm, examination of individual color-coded distance maps revealed significant disparities between the simulated and the actual results.

## Introduction

A dental setup is a procedure that virtually corrects a patient’s malocclusion. It is considered an important diagnostic tool to assist treatment planning [1]. The traditional dental setup (TS), introduced by Harold Kesling, involves separating plaster crowns from the dental cast and rearranging them through placement in dental wax [2]. Yet, the TS technique is time-consuming and requires expertise, laboratory procedures, and thus, increased costs [3]. On the other hand, the three-dimensional (3D) digital dental setup (DS) is performed on a personal computer using software applications to modify 3D digital dental models that are nowadays readily available [4–6]. The latter offer reliable 3D information on intraoral surfaces and are also easy to use and convenient for patients [4–6].

The DS offers the advantages of fast construction, higher precision, since there is no material loss during tooth crown separation, and unlimited attempts. It also allows for the selection of dental arch-forms from online libraries or for custom creation [2, 7]. An additional advantage of the DS derives from the ability to superimpose corresponding dental models during the process, allowing for useful comparisons of the original to the predicted model [8]. The analysis of differences in tooth position requires stable structures as superimposition references [9], such as the middle 2/3 of the third rugae in the maxillary cast, as well as various other superimposition principles to be taken into account during implementation and outcome interpretation [3, 10–13].

Recently, DS has been employed to review proposed orthodontic treatment plans by sharing them with orthodontists and other healthcare providers [14]. Additionally, DS has been utilized to present treatment options to patients and their parents, enabling them to comprehend the available choices, and participate in the development of the definitive treatment plan [15, 16]. In this manner, DS facilitates treatment planning and enhances communication between patients and doctors, as well as among healthcare professionals [17, 18].

Despite the various advantages and wide-ranging applications of DS, previous research has primarily concentrated on assessing its accuracy in simulating aligner treatment outcomes [1, 19, 20]. There is still a lack of evidence on the reliability of DS regarding the prediction of fixed orthodontic treatment outcome. Among others, DSs can be particularly helpful in borderline cases (e.g., class I with missing laterals or crowding cases that can be treated with and without extractions), as well as in multidisciplinary restorative cases or in orthognathic surgery cases where it is crucial to determine teeth positions due to orthodontic tooth movement pre- as well as post-surgery. Since the DS’s accuracy in buccal fixed appliances has not been adequately tested so far, we decided to perform this investigation in relatively simple cases, where the treatment components could be standardized and better simulated. Therefore, this study aimed to evaluate the accuracy of the prediction of the fixed appliance treatment outcome through 3D digital setups, in patients with class I malocclusion and moderate crowding. For this, digital dental models representing the predicted outcome were superimposed to the actual treatment outcome models.

## Materials and methods

### Study design and participants

This study is a prospective, one-group diagnostic trial, conducted at the Department of Orthodontics, *University of Damascus, Syria*. The ethical approval for the study was obtained from the Local Research Ethics Committee of the Faculty of Dentistry, University of Damascus (Ref no. DN-290122-20) before trial commencement. All participants signed an informed consent form for participation in this trial.

The eligibility criteria aimed to include patients between 18 and 24 years of age, with class I malocclusion (molar and canine relationships deviated ≤ 1/4 of a cusp from Class I occlusion and ANB between 0 and 4°) and normal overbite (approximately one-third coverage of the mandibular incisors’ clinical crown length or 1.6 to 3.2 mm), combined with moderate crowding (Little’s irregularity index: 4–6 mm), upper incisor angle (U1) to SN ≤ 104°, lower incisor angle (L1) to GoMe ≤ 92°, good oral hygiene, and good periodontal health (visual inspection and clinical examination). The exclusion criteria were previous orthodontic treatment, bimaxillary dentoalveolar protrusion, congenitally missing or extracted teeth (except for the third molars), and treatment plans including tooth extractions.

**Fig. 1:**
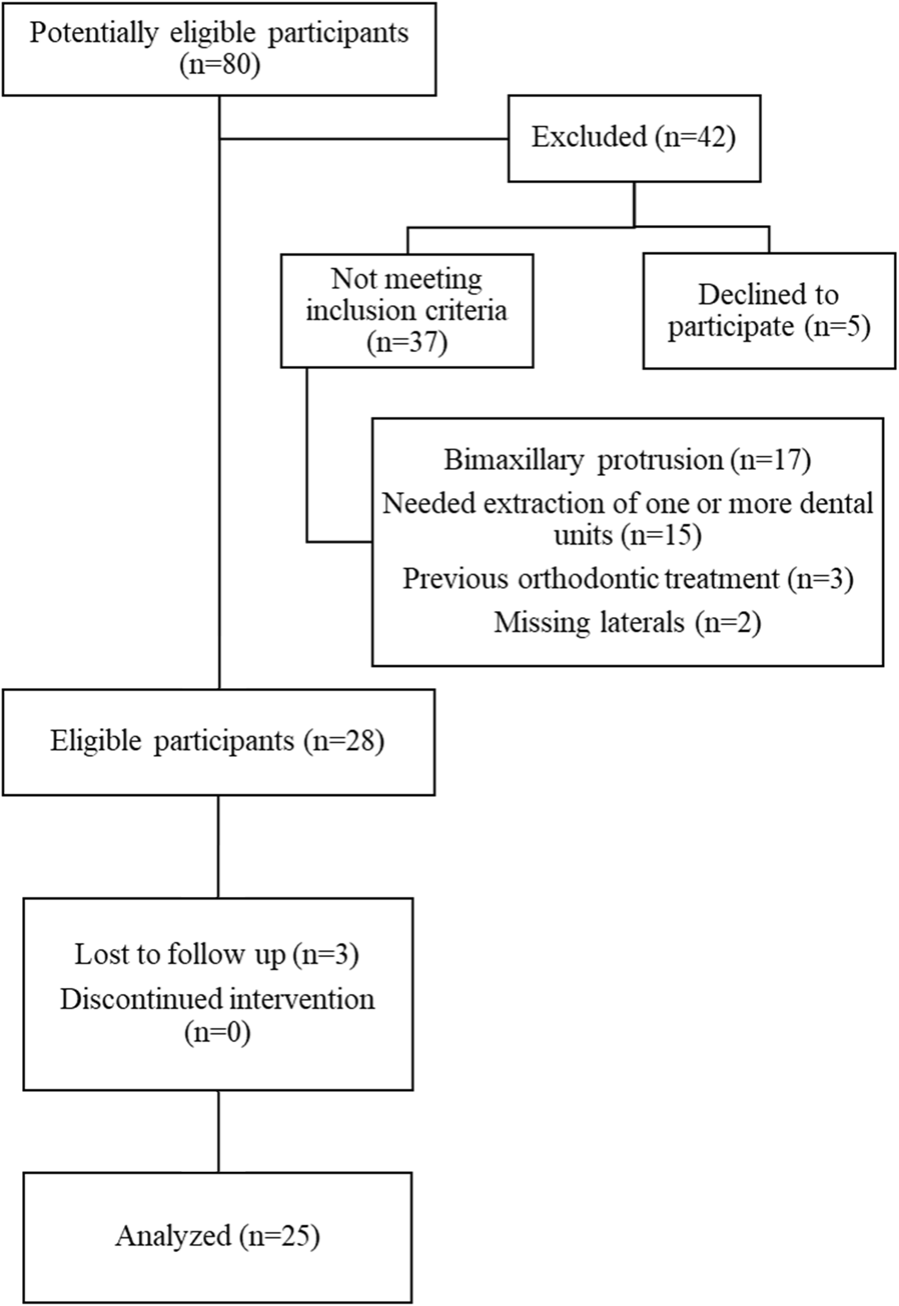
Participants’ flow diagram

All patients who had been referred to the university clinic with an initial clinical diagnosis of moderate dental crowding between December 2020 and December 2021 were assessed for eligibility. From 80 patients that were initially considered, thirty-three were eligible and five of them declined to participate. Thus, 28 patients were enrolled after distributing information sheets and collecting signed written informed consent forms (Fig. 1).

An a priori sample size calculation was not conducted for this research due to the lack of existing data, the type of the study, and its primary outcome, which was descriptive. All individuals meeting the eligibility criteria during the assessment period were enrolled aiming to achieve a minimum sample of 20 patients. An adequate sample size was achieved, to generate representative and meaningful empirical evidence as indicated by previous relevant studies [1, 19, 20].

For each participant, after collecting the standard diagnostic records (cephalometric and panoramic radiographs, intra/extra-oral photographs, and initial dental casts), a thorough case study was held and a detailed treatment plan was formulated, including the determination of the required amount of interproximal reduction (IPR) and the selection of the proper archwire form to be used.

### Digital model acquisition

Pre- and post-treatment alginate impressions were taken (Cavex^©^ CA37, CAVEX, RW Haarlem, the Netherlands) and immediately poured with plaster (chromotypo4, Lascod^©^ S.p.A, Florence, Italy). After that, all plaster casts were scanned using a high-accuracy extra-oral scanner (phase-shifting optical triangulation/LED 150 ANSI-lumens, accuracy 4 μm, Laboratory scanner T710 Medit^©^, Seoul, Korea) and transformed into digital models of Stereolithography (STL) format (initial digital model: IDM, final digital model: FDM). Each maxillary 3D mesh consisted of approximately 20,000 vertices, and each mandibular 3D mesh consisted of approximately 18,000 vertices.

### Digital setup generation process

A semi-automatic virtual setup method was applied in each patient’s IDM through Orthoanalyzer software (version 19.1, 3Shape®, Copenhagen, Denmark). The mid-sagittal, the horizontal and the vertical planes were defined in the models, virtual bases were constructed, and the teeth were segmented. Afterward, virtual brackets were placed at the facial axis points, according to the MBT® bracket placement standards, and buccal tubes at the first molars (MBT® 0.022 inch standard metal brackets with hooks on canines, first, and second premolars; American Orthodontics, Sheboygan, USA). Interproximal reduction (IPR) was performed according to the case-specific treatment plan, by using software tools. The linear mesiodistal dimension of each affected tooth crown was measured to confirm the accurate application of the planned IPR amount. Because all patients had ovoid dental arch-forms, a 0.019 × 0.025 inch stainless steel ovoid-shaped archwire (American Orthodontics, Sheboygan, USA) was selected to align the teeth, since this would be the final archwire in the actual treatment process. This was automatically placed at the same distance from the first molars on both sides and the middle point was set on the dental midline. Eventually, a predicted digital model (PDM) was created.

### Provided orthodontic treatment

The first author provided the orthodontic treatment for all patients by bonding metal labial brackets on both jaws at the same appointment and buccal tubes on first molars (MBT® 0.022 inch slot, American Orthodontics, Sheboygan, USA). The dental arch alignment stage was achieved through the following archwire sequence: 0.012 inch, 0.014 inch, 0.016 inch, 0.016 × 0.022 inch, and 0.017 × 0.025-inch Nickel-Titanium wires, followed by 0.017 × 0.025 inch and 0.019 × 0.025-inch stainless steel wires (American Orthodontics, Sheboygan, USA). Patients were controlled every 4 weeks and, if possible, the archwire was changed. Rebonding of individual brackets was performed at any stage to account for errors in bracket positioning as needed. No intermaxillary elastics were used. The amount of IPR, defined during the treatment plan, was performed utilizing separating strips (Horico®, Berlin, Germany). IPR was performed in an individualized manner depending on the needs of each case.

When the Little’s irregularity index equaled zero, and a normal overjet (2–3 mm) and overbite (1/3 of lower incisors’ clinical crown length) were achieved, the treatment was considered finished. The fixed appliances were removed and four final alginate impressions were obtained (two maxillary and two mandibular) and poured with plaster within 1 h. Two of the subsequent dental casts were used to create vacuum-formed retainers and the other two to produce the final dental models (FDM).

### Superimposition procedure

The two digital model sets (PDM, and FDM) of each patient were imported in Viewbox 4 software (version 4.1.0.1 BETA, dHAL Software, Kifissia, Greece) and colored differently to facilitate processing. Three superimposition reference areas were selected on the PDMs. One consisted of the middle 2/3 of the second and third rugae and the area 5 mm dorsal to them [3, 4, 10–12] and two other of the upper and the lower dental arches (teeth 16 to 26 and 36 to 46, respectively), to assess dental arch differences regardless of the surrounding structures. Digital 3D copies of the reference areas were created to assess the potential differences of the PDM from the FDM after superimposition. Consistently, the FDM retained its position in space and the PDM was approximated to it.

Three best fit superimposition procedures were performed: (1) superimposition of the maxillary PDM and FDM on the palatal reference area, to measure differences in the predicted from the final dental arch position within the jaw, (2) superimposition of the maxillary teeth of the PDM and the FDM, to compare the final dental arch predicted by the DS to the actual treatment outcome, and (3) same as 2, but regarding the mandibular arch. The superimposition settings were defined according to Vasilakos et al. [12] and were: 100% estimated overlap of meshes, matching point to plane, exact nearest neighbor search, 100% point sampling, 50 iterations, exclude overhangs.

After each superimposition, color-coded distance maps were generated to visualize and quantify differences among the compared surface models.

### Outcome measures

Following each superimposition, the distances between the corresponding closest points of interest at the approximated surface areas were exported from Viewbox 4 software and imported in Microsoft Excel data sheets (Microsoft Corporation, Redmond WA, USA). The mean absolute distances (MAD) and the standard deviations of the absolute distances (SDAD) comprised the metrics to assess the study outcomes, which were:

- The MAD and the SDAD of the predicted from the final maxillary dental arch, following superimposition on the palatal reference area.
- The MAD and the SDAD of the predicted from the final maxillary dental arch, following superimposition on the dental arch.
- The MAD and the SDAD of the predicted from the final mandibular dental arch, following superimposition on the dental arch.
- The MAD and the SDAD of the predicted from the final palatal reference area, following superimposition on it.

### Visual assessment of color-coded distance maps

In the respective color maps, the red color represents the maximum positive value, indicating that the shown model is closer to the viewer than the reference model, the green describes the near zero mm value (perfect match), and the blue color describes the minimum negative value, indicating that the shown model is further from the viewer than the reference model. Visual assessment of the color-coded distance maps was performed by the first author as described below. Three different groups of teeth were evaluated within each dental arch: the anterior teeth (from canine to canine), the premolars, and the first molars. Each group’s buccal, palatal/lingual, and incisal/occlusal surfaces were assessed, the directions of differences between the compared surfaces (PDM versus FDM) were recorded, and the outcomes were grouped. The assessments were reviewed and confirmed by the last author, with any disagreements resolved through consensus.

### Intra-operator reproducibility of the superimposition outcomes

The entire superimposition process was repeated by the same investigator on ten randomly selected cases, one month after the initial assessment, to test intra-operator reproducibility.

### Statistical Analysis

The statistical analysis was performed by using the IBM SPSS statistics for Windows (Version 28.0. Armonk, NY: IBM Corp). Data were tested for normality using the Shapiro–Wilk test and showed normal distribution. Thus, parametric descriptive and comparative statistics were applied.

Paired and unpaired Student’s t tests were used for comparative analysis regarding superimposition outcomes. The mesiodistal dimensions of teeth who underwent IPR during DS creation were compared to the actually reduced teeth during treatment, on ten randomly selected cases, through a paired t test.

Bland Altman’s plots were used to test the intra-operator reproducibility on the superimposition outcomes and boxplots to show the differences between corresponding assessment areas on the superimposed models.

The level of significance for the study was set at 0.05.

### Data availability

The datasets generated and/or analyzed during the current study are available from the corresponding author upon reasonable request.

## Results

### Patient sample

Twenty-eight young adult patients (18 females, 10 males; mean age: 20.68, SD: 1.91 years) participated in this trial. Three patients withdrew from the study during the orthodontic treatment, because they moved to another country. Eventually, 25 patients were analyzed. Table 1 describes the basic characteristics of the study sample.

**Table 1:** Descriptive statistics of basic sample characteristics

	Mean (SD)		Minimum		Maximum	
Age at T0 (years)	20.3 (1.7)		18.0		24.0	
Treatment duration (months)	11.5 (1.3)		9.0		13.0	

	T0	T1	T0	T1	T0	T1
Overjet (mm)	2.6 (0.3)	2.7 (0.3)	1.8	2.1	3.17	3.42
Overbite (mm)	2.3 (0.5)	2.2 (0.3)	1.6	1.6	3.2	2.8

*SD* Standard deviation, *T0* Before treatment, *T1* After treatment

### Outcome assessment

#### Predicted versus final maxillary dental arch

**Fig. 2:**
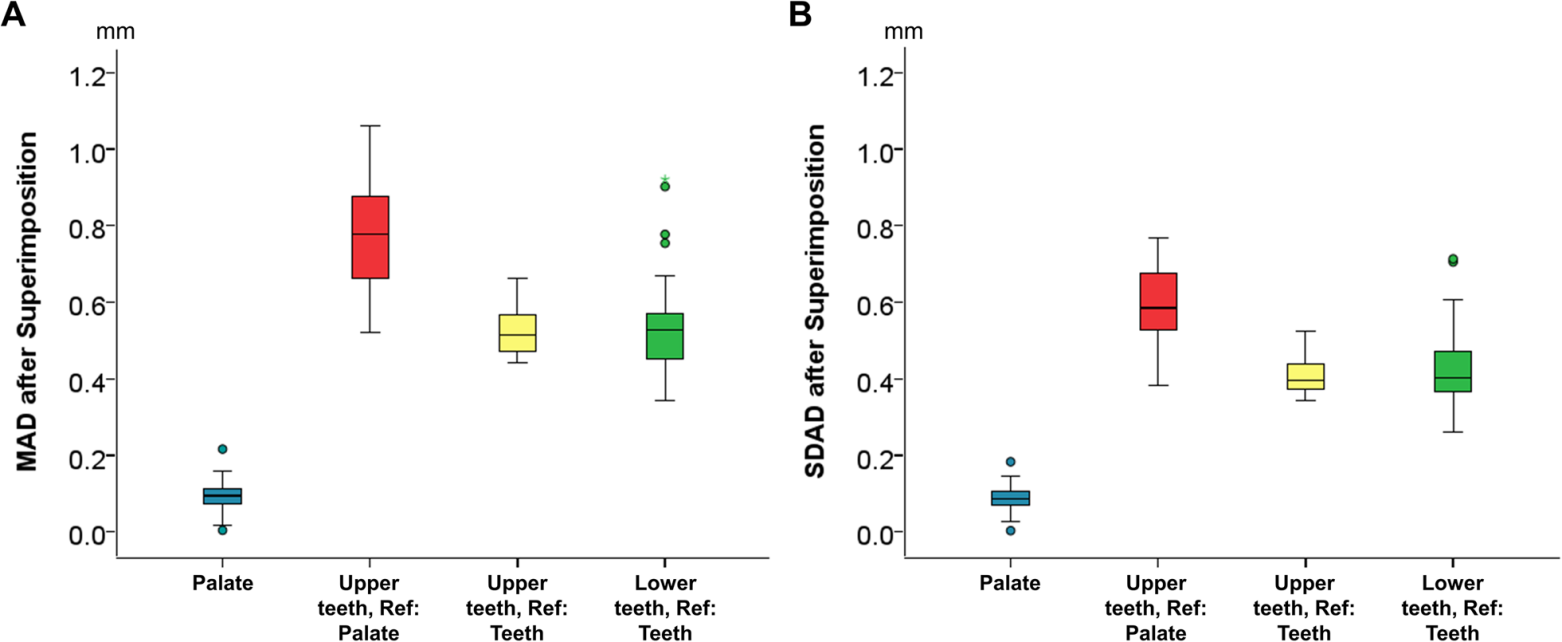
Boxplots showing the (**A**) MAD and (**B**) SDAD between the superimposed predicted and final models on the reference areas and regarding the outcomes described on the horizontal axis. The SDAD values represent the standard deviations of the absolute distances between superimposed, corresponding surface meshes of each patient that consist of thousands of points/distances, thus indicating within mesh/patient variation and not the variation between patients. Ref.: Superimposition reference. mm: millimeter

**Fig. 3:**
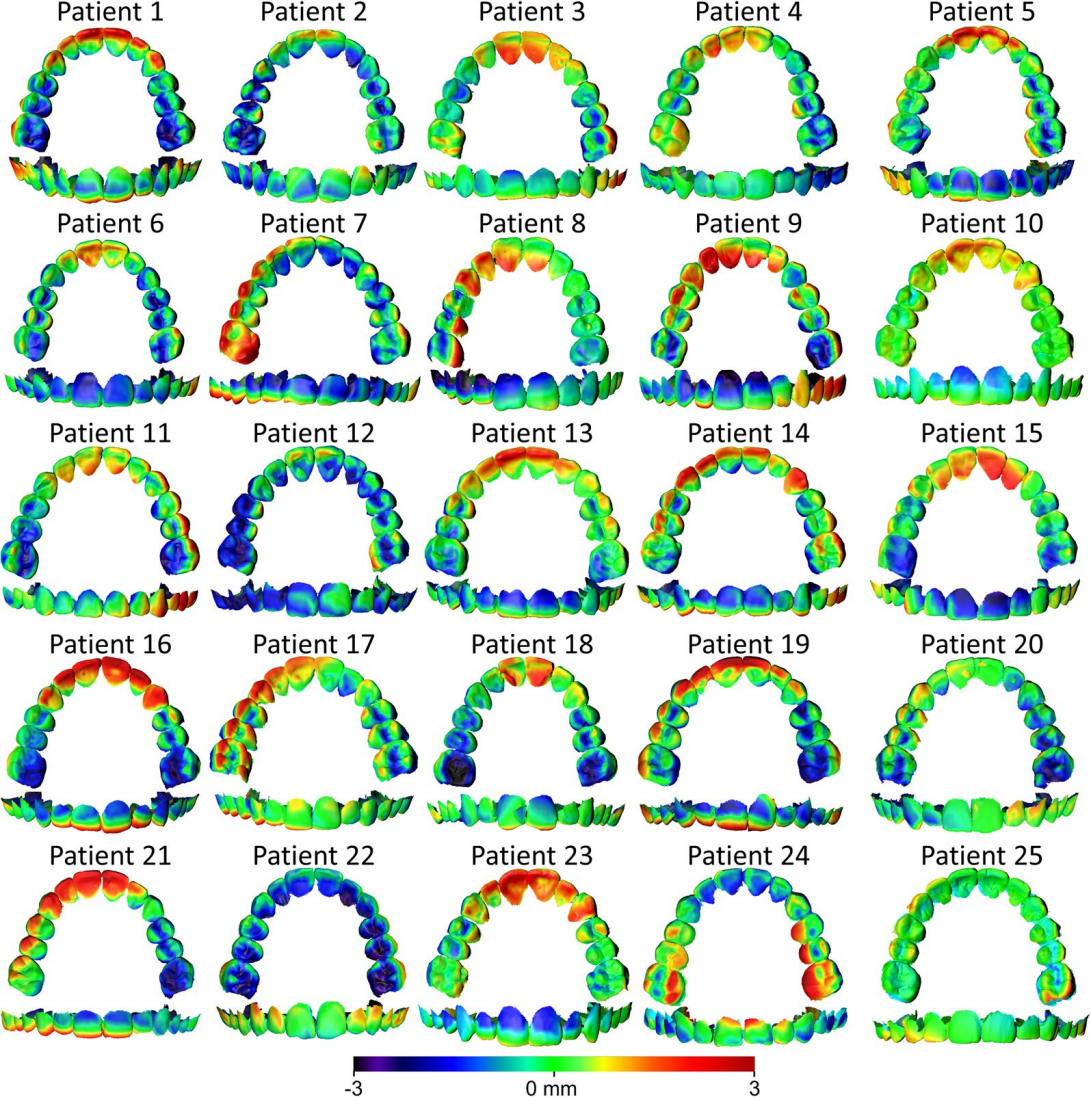
Color-coded distance maps generated following the superimposition of the maxillary PDM and FDM on the palatal reference area (shown model: PDM, reference model: FDM). The upper anterior teeth, premolars, and first molars were accurately positioned at the digital setup in 5, 2, and 2 cases, respectively (error within 0.5 mm). There was no specific error pattern regarding tooth position in 8, 23, and 13 cases, respectively, where the errors were evenly distributed over the dental arch. In six cases, the upper anterior teeth were positioned more palatally than the actual treatment outcome (by approximately 3 mm), while in six other cases, they exhibited greater palatal inclination. In eight cases, the first molars exhibited a more buccal position than the actual outcome, and in a few cases, they showed greater buccal inclination

The MAD of the predicted from the final maxillary dental arch was 0.77 ± 0.13 mm (SDAD: 0.59 ± 0.10 mm), following superimposition on the palate (Fig. 2). The respective color-coded distance maps (Fig. 3) revealed that the predicted individual tooth positions matched accurately the final treatment outcome in a few cases only. Half of the digital setups predicted a more palatal position and inclination of the upper anterior teeth, whereas the first molars appeared buccally positioned or inclined compared to the actual outcome. No specific pattern was evident in the premolars. The rest of the prediction models also presented errors within the dental arch, sometimes reaching 3 mm, but without any spatial pattern.

**Fig. 4:**
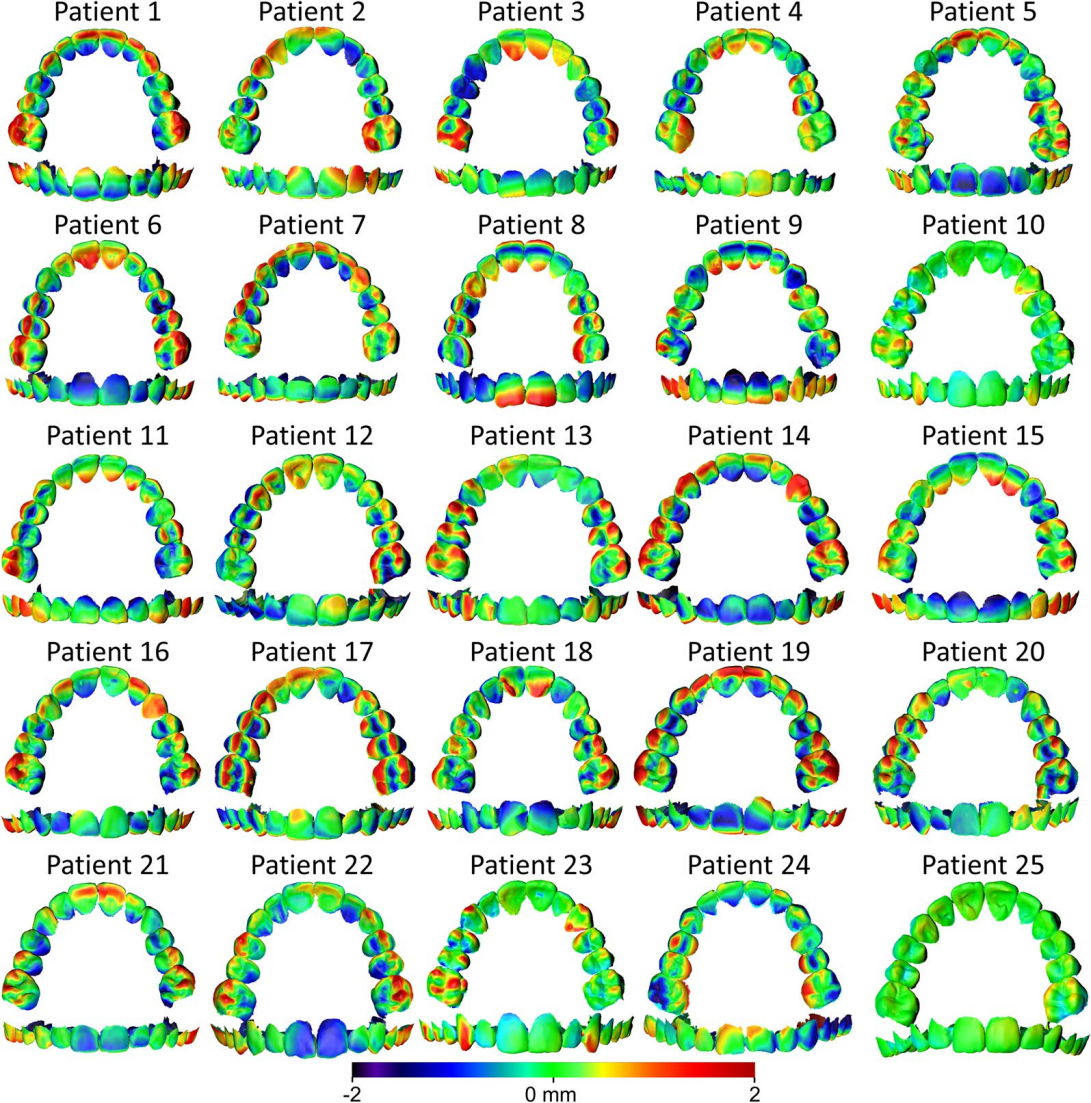
Color-coded distance maps generated following the superimposition of the maxillary PDM and FDM on the maxillary dental arch (shown model: PDM, reference model: FDM). Among the upper anterior teeth, premolars, and first molars, accurate positioning was achieved in 7, 4, and 3 cases, respectively (error within 0.5 mm). There was no distinct tooth positioning error in 10, 12, and 16 cases, respectively, where the errors were evenly distributed over the dental arch. In five cases, the upper anterior teeth were positioned more palatally than the final treatment outcome (by approximately 2 mm), and in a few cases, they exhibited a greater palatal inclination. In five cases, the premolars were positioned more buccally, while in five other cases, they exhibited a more palatal position in the PDM. The first molars were buccally positioned in a few cases, whereas in three cases, they showed a more palatal position compared to the FDM

The MAD of the predicted from the final maxillary dental arch was 0.52 ± 0.06 mm (SDAD: 0.40 ± 0.05 mm), following superimposition on the maxillary dental arch (Fig. 2). Analysis of color-coded distance maps (Fig. 4) unveiled that the predicted positions of individual teeth matched precisely the actual treatment outcome in a limited number of cases. Approximately, half of the digital setups simulated a more palatal inclination and position of the upper anterior teeth and a more buccal position and inclination of the premolars and the first molars, as compared to the actual outcome. The remaining predicted models also displayed errors up to 2 mm within the dental arch, without any repeated pattern.

Paired *t* tests showed statistically significant differences between the MADs obtained following superimposition on the palate versus that on the dental arch (Mean difference: 0.245 mm, *P* < 0.001), as well as for the respective SDADs (Mean difference: 0.181 mm, *P* < 0.001) (Fig. 2).

#### Predicted versus final mandibular dental arch

**Fig. 5:**
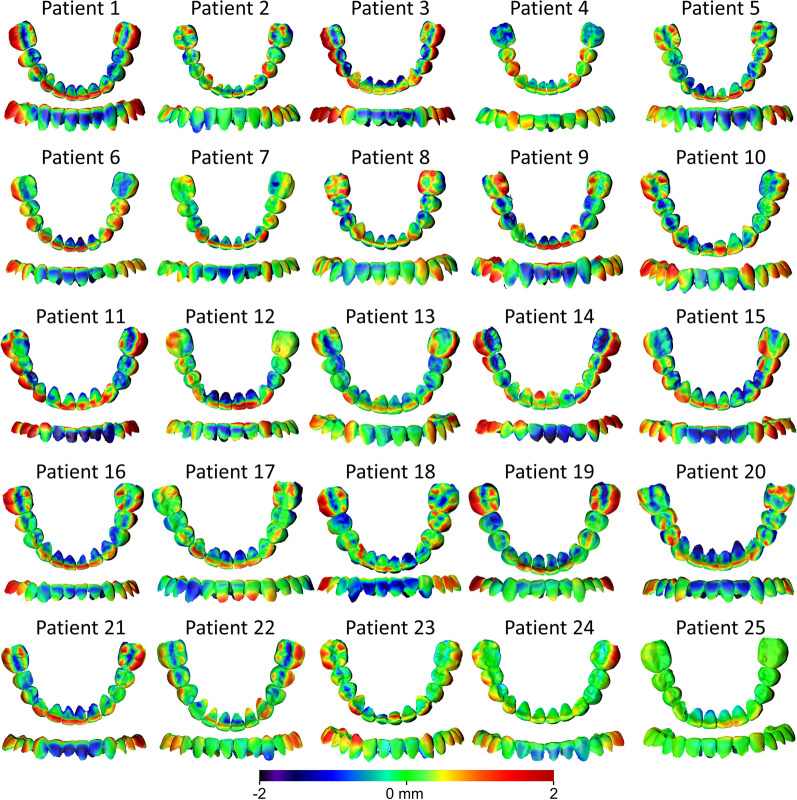
Color-coded distance maps generated following the superimposition of the mandibular PDM and FDM on the mandibular dental arch (shown model: PDM, reference model: FDM). The lower anterior teeth, premolars, and first molars were accurately positioned in 3, 5, and 5 cases, respectively (error within 0.5 mm). There was no specific pattern in 9, 13, and 4 cases, respectively, where the error was distributed over the dental arch. In half of the cases, the lower anterior teeth had more lingual inclination than the actual outcome. In the PDM, the premolars exhibited more buccal inclination in seven cases and the first molars were more buccally positioned (by approximately 2 mm) in 16 cases

The MAD of the predicted from the final mandibular dental arch was 0.55 ± 0.15 mm (SDAD: 0.42 ± 0.11 mm), following superimposition on the mandibular dental arch (Fig. 2). There were no significant differences between the MADs and the SDADs measured on the maxillary versus those on the mandibular dental arches following superimposition on the dental arches (MAD Mean difference: − 0.032 mm, *P* = 0.326; SDAD Mean difference: − 0.017 mm, *P* = 0.492). The respective color-coded distance maps (Fig. 5) demonstrated that the predicted mandibular tooth positions were in accordance with the actual outcome in limited cases. Approximately, half of the digital setups predicted more lingual position and inclination of the lower anterior teeth than the actual outcome. On the contrary, the premolars and the first molars were positioned and inclined more buccally. The remaining predicted models also exhibited within-arch discrepancies up to 2 mm, with no discernible pattern.

#### Initial versus final palatal reference area

**Fig. 6:**
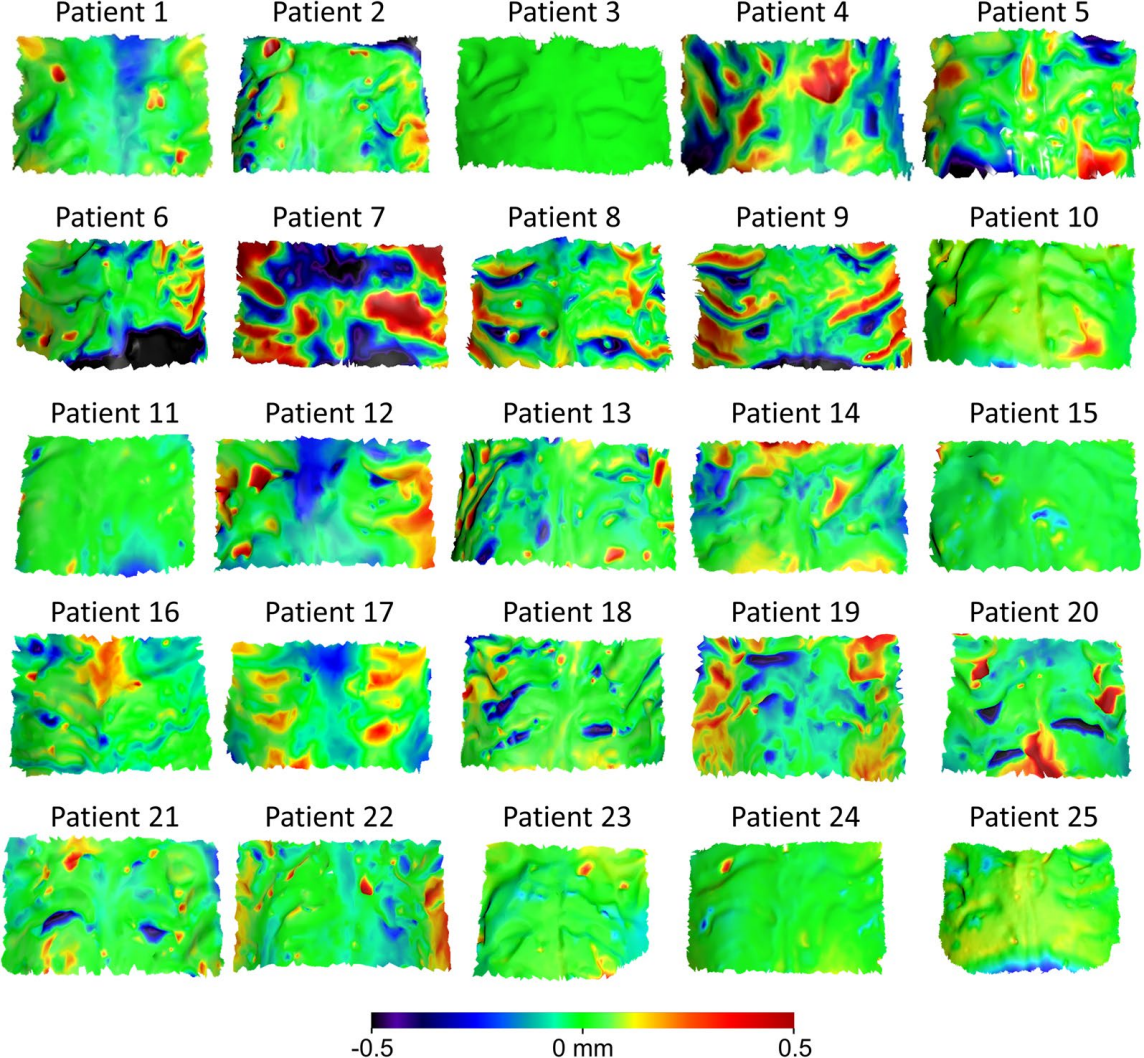
Color-coded distance maps showing the differences in the palatal reference area, following the superimposition of the maxillary PDM and FDM on the same area (shown model: PDM, reference model: FDM). Most of the cases showed morphological stability, whereas a few cases (patient 4, 7–9, 12 19) presented slight variations (within 0.5 mm), indicating a flattening of the palate in the FDM

The MAD of the palatal reference area of the PDM, which was identical to that of the IDM (unaffected by the DS process), from the FDM was 0.09 ± 0.04 mm (SDAD: 0.08 ± 0.03 mm), following superimposition on the palatal reference area (Fig. 2). Visualization of color-coded distance maps (Fig. 6) indicated that the palatal reference area was anatomically stable, showing minimal changes (within 0.5 mm). Slight changes were evident in six cases toward a flattening of the palate in the FDM as compared to the PDM/IDM.

### Intra-operator reproducibility

**Fig. 7:**
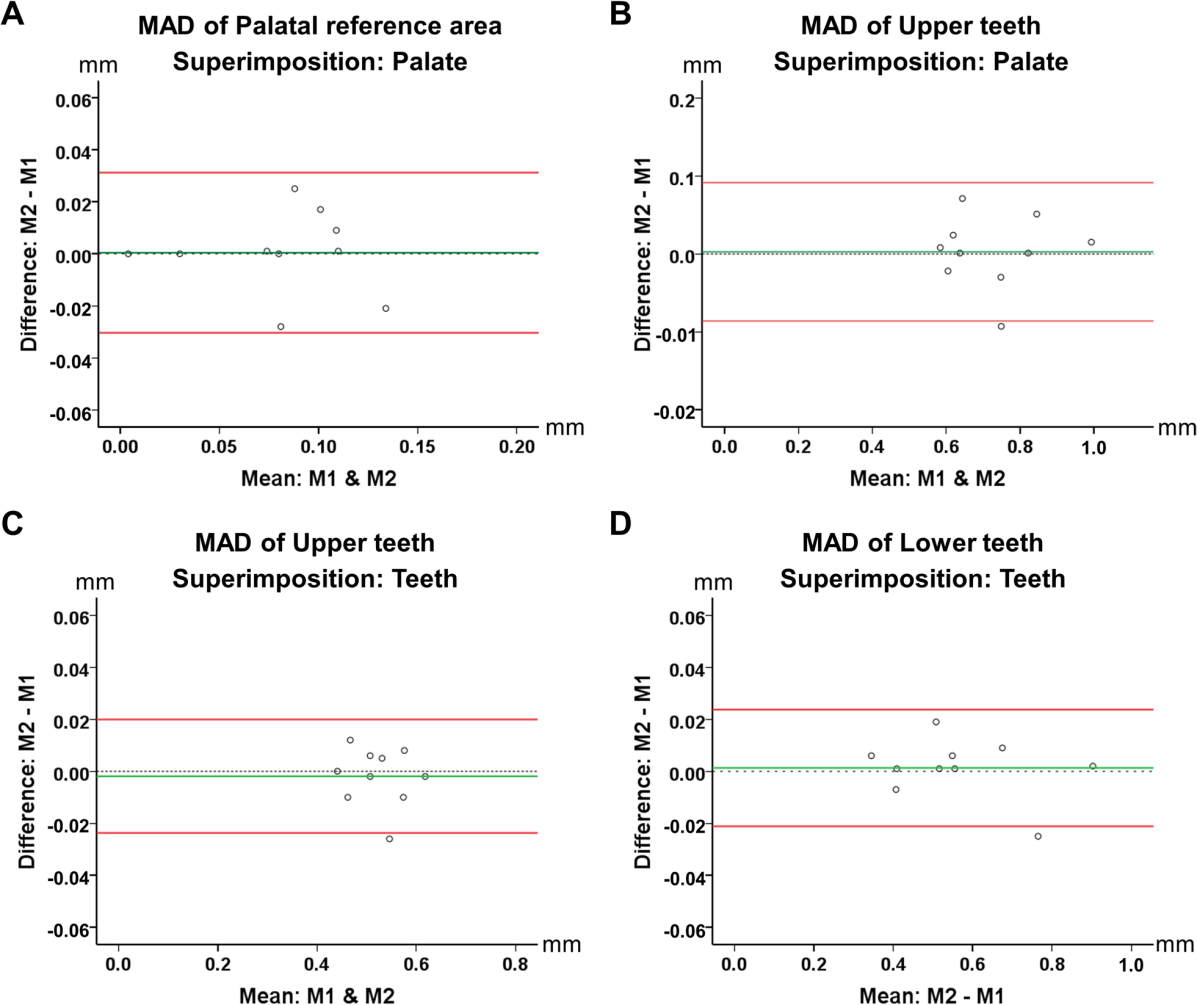
Bland–Altman plots showing the intra-operator reproducibility of the outcome assessment process. The measured variable was the MAD between the superimposed PDM and FDM models on (**A**) the palate, (**B**, **C**) the maxillary dental arch, and (**D**) the mandibular dental arch. The axes lengths represent the true range of the measured MAD values. The continuous horizontal green line shows the mean reproducibility of each outcome and the continuous horizontal red lines demonstrate the 95% limits of agreement. The dashed horizontal line indicates the 0 level, and thus, perfect reproducibility. M: Measurement. mm: millimeter

The intra-operator reproducibility of the calculated outcome measures was high. The average error was in all cases almost zero, the individual measurement error remained consistently within 0.1 mm, and there was no evidence that it was increasing by an increase in the magnitude of the original measurement. The respective Bland–Altman plots are shown in Fig. 7.

### Accuracy of digital teeth interproximal reduction

The width of the teeth that were subjected to digital and actual IPR was measured in the PDM and FDM models of ten randomly selected cases, respectively. Paired t test showed no statistically significant differences between the respective tooth widths (Mean difference: 0.18 ± 0.10 mm, *P* = 0.216).

## Discussion

Utilizing DS may significantly improve the ability to communicate expected treatment results to patients and facilitate information exchange among dental specialists regarding treatment plans. Therefore, it is crucial to evaluate the accuracy of DS in depicting the anticipated treatment outcomes. Previous studies have investigated the accuracy of achieving the predicted tooth movements by using clear aligners or pre-adjusted lingual appliances [21–23], while others have studied the arch-form prediction or the consistency in treatment plan formulation between traditional and digital methods [18, 24]. The present study is the first to examine the accuracy of a 3D DS method that simulates the orthodontic treatment outcome produced by fixed labial appliances, using the straight-wire technique, namely the most widely used technique in daily practice. The assessment was based on the 3D superimposition of patients’ predicted with final treatment models.

The studied malocclusion was Angle Class I combined with moderate crowding and no excessive labial protrusion of the incisors, overjet, and overbite. This is a common malocclusion type for which patients seek treatment [25]. Additionally, such cases are usually treated by leveling and alignment with the aid of IPR, with a minimum requirement of sagittal and vertical tooth movements and minor or no need for intermaxillary elastics. This allowed us to closely simulate the actual treatment process during the DS (e.g., same brackets, final archwire, and amount of IPR), which might have aided in minimizing confounding and technique-sensitive errors. This focused our assessment on the accuracy of the leveling and alignment simulation.

The software as well as the 3D superimposition and outcome assessment methods applied in the study have been widely tested previously and proved reliable by different operators [6, 10–12]. We selected the entire dental arch as superimposition reference area to give equal weight on all dental arch areas. If the superimposition was performed on the anterior teeth, this would result on a better best fit there, but on higher errors on the posterior teeth, confounding the outcomes [4]. In the present study, the intra-operator reproducibility regarding superimposition outcomes was very high in accordance with previous reports [6, 10–12]. Moreover, when comparing the digital IPR with the actual IPR amount, no statistically significant differences were observed. The amount of tooth reduction defined during treatment planning was meticulously applied by conducting linear measurements following each IPR session to ensure that it remained within the specified limits. This is in accordance with a previous study [26] reporting that the digitally planned IPR can be accurately applied clinically.

An a priory sample size calculation was not performed since the main outcome was descriptive, the patients were not subjected to any risks when participating in this study, and there were no existing data for the secondary outcomes. Post hoc analysis was performed for the paired Student’s t tests using G*Power software (version 3.1.9.2; Heinrich-Heine-University, Düsseldorf, Germany) under the following assumptions: the alpha level, effect size and study power were set at 0.05, 0.5, and 0.9, respectively. The variable of interest was the MAD of the upper dental arch of the PDM from the FDM after superimposition on the palate, compared to the MAD after superimposition on the upper dental arch. The standard deviations for the studied pairs were 0.13 and 0.06 mm, respectively, and the analysis revealed that 18 patients were required. Regarding the MAD of the upper dental arch of the PDM from the FDM, compared to the MAD of the lower dental arch, after superimpositions on the dental arches, the standard deviations for the studied pairs were 0.06 and 0.15 mm, respectively, and the analysis revealed that 22 patients were required. For both outcomes, the sample size was sufficient.

There were statistically significant differences in the recorded MAD of the upper PDM from the FDM after superimposition on the palate compared to superimposition on the dental arch. This was anticipated for two reasons. At first, when superimposing on the palate, any alteration in the spatial relations between the dental arch and the palate, occurring between the initial (in PDM the palate remains intact) and the final model, is factored into the evaluation of the dental arch outcome. Hence, these differences are added to any localized differences in the dental arches, due to DS imprecision. The magnitude of this effect would have been more pronounced in cases of greater tooth displacement or extended treatment periods, particularly in individuals with active growth [5, 11, 27]. The second reason is that when the superimposition reference area is located at a distance from the assessment area, small rotational differences between the superimposed reference areas have an increased impact on the outcomes [28]. On the contrary, there were no differences between the maxillary and mandibular MADs of the superimposed PDM and FDM dental arches, indicating similar inaccuracies of the DS process in both jaws, compared to the actual treatment result.

In almost half of the cases the predicted treatment outcome was characterized by posteriorly wider upper and lower dental arches (buccally placed first molars) and palatally/lingually positioned or inclined anterior teeth. The DS matched adequately the final treatment result in few cases only, whereas the remaining cases exhibited unique variations without distinct recurring patterns. Although most treatment components were precisely incorporated into the DS generation process, limitations that could explain the detected inaccuracies still exist. Apart from the complexity of the continuously changing forces and moments generated by the full fixed appliance system, until now, no available software utilizes algorithms that can accurately simulate the cellular response to these. The role of the periodontal ligament, a significant factor influencing orthodontic tooth movement, remains largely elusive, complicating the 3D DS simulation process [29]. Another puzzling factor might be the perioral muscular envelope, which could modify the applied orthodontic forces and thus impact the treatment outcomes [30].

Several studies reporting on the efficacy of clear aligners showed inconsistencies between the predicted and the achieved treatment outcomes, although these are not comparable to our study primarily due to fundamental differences in the applied treatment means [19, 31–33]. In aligner treatment, the appliances are constructed according to the targeted outcomes, whereas we intended to predict the produced outcomes by the prefabricated fixed orthodontic appliances. Studies on DS with fixed orthodontic appliances also indicated clinically significant inaccuracies, but these also present major differences to our study. Two of these studies [22, 34] investigated the accuracy of simulating the orthodontic phase’s outcome in orthodontic-surgical cases, but they both reported on setups where tooth position was defined by the operator. Two other studies investigated the efficacy of lingual orthodontic appliances showing promising results with higher inaccuracies at the buccolingual position of the posterior teeth [23, 35]. As with aligners, the tested lingual appliances were also customized. There is also one study on labial fixed appliances that tested the efficacy of a customized CAD/CAM bracket system on a sample with similar characteristics to ours. Also this study showed that the transversal position of the posterior teeth and the anteroposterior position of the central incisors are less predictable [36]. The latter is often evident in several of the aforementioned studies, as it was in our study, despite the fundamental differences in treatment approaches, samples, and methods.

Although there is no study in the literature that can be directly comparable to ours, an important drawback of the relevant studies is that conclusions are primarily based on average measures. Our study underscores the significance of evaluating individual cases rather than relying solely on group-based analyses in diagnostic accuracy studies. In many situations, including the current study, using average measures alone may not adequately capture important random errors, as they primarily focus on the absence of systematic errors. In simpler terms, while the average error might be minimal or close to zero, a prediction method intended for clinical use should be effective for every individual case, not just when examined as an average across a group. Hence, it is essential to assess individual errors to validate the prediction method and assess its reliability for every single case. This principle applies to both individual case analyses and evaluations within the same individual. The latter aspect involves visualizing color-coded distance maps, which reveal errors across the entire surface. This step is vital because average measures within individuals, such as MAD, can potentially mask notable inaccuracies at localized sites, which could have significant clinical implications. Future research should also assess the traditional setup procedures in this manner, since solid evidence to support their application is still lacking.

### Limitations

This study included patients with mild-to-moderate Angle Class I malocclusion. Perhaps higher DS errors would have been evident in more severe cases, since the planned tooth movements would be larger. Additionally, bracket placement was performed directly by the orthodontist, which might have caused variation between the actual and the digital bracket placement. A single software was used to create the DS, and thus, the results cannot be generalized to other software applications. Future research should focus on the simulation of orthodontic treatment outcomes for other types of malocclusions, consider indirect bracket placement method, and test different software.

## Conclusions

A 3D digital simulation of orthodontic treatment outcomes using buccal fixed appliances and the straight-wire technique proved inaccurate for moderate crowding cases. Discrepancies between predicted and actual outcomes were often within 2–3 mm, reaching clinically significant levels. Only a minority of cases exhibited close predictions (within 0.5 mm), with half of the sample displaying broader posterior dental arch width and more palatal/lingual positioning and inclination of anterior teeth compared to the final result.

## Data Availability

The datasets generated and/or analyzed during the current study are available from the corresponding author on reasonable request.

## Abbreviations

TS: Traditional setup
DS: Digital setup
3D: Three-dimensional
IDM: Initial digital model
PDM: Predicted digital model
FDM: Final digital model
IPR: Interproximal reduction
VFR: Vacuum-formed retainers
MAD: Mean absolute distances
SDAD: Standard deviation of the absolute distances
OTM: Orthodontic tooth movement
